# Evaluation of Availability of Survey Data About Cannabis Use

**DOI:** 10.1001/jamanetworkopen.2020.6039

**Published:** 2020-06-10

**Authors:** Kimberley H. Geissler, Kia Kaizer, Julie K. Johnson, Samantha M. Doonan, Jennifer M. Whitehill

**Affiliations:** 1 Department of Health Promotion and Policy, University of Massachusetts Amherst School of Public Health and Health Sciences, Amherst; 2 Massachusetts Cannabis Control Commission, Worcester

## Abstract

**Importance:**

In response to increasing public support for cannabis legalization, understanding associations of state and federal policy changes related to cannabis legalization with patterns of cannabis use is important. A challenge for public health monitoring and research is significant variation in data availability related to cannabis use behaviors and perceptions across and within states and over time, including the availability of prelegalization vs postlegalization data.

**Objective:**

To review data available on cannabis use and related behaviors over time in Massachusetts and the US.

**Design, Setting, and Participants:**

This case series examined state and national surveys on public health and related behaviors and outcomes to review availability of cannabis-related data for Massachusetts for 8 key indicators over time. Additionally, the Behavioral Risk Factor Surveillance System (BRFSS) and Youth Risk Behavior Surveillance System for all states were reviewed. The analysis was conducted between February 1, 2019, and March 18, 2020.

**Exposures:**

Surveys that enable state-level estimation of cannabis use and related behaviors.

**Main Outcomes and Measures:**

Eight key indicators related to cannabis use behaviors and perceptions: lifetime cannabis use, age of initiation, frequency of use, location of use, method of use, source of cannabis, perceptions of cannabis, and reason for use (ie, medical vs nonmedical).

**Results:**

There were 7 surveys that monitored cannabis use and related behaviors in Massachusetts for adolescents and adults. No surveys monitored all 8 indicators of interest, and availability over time was limited. In the most recent BRFSS, 24 states asked cannabis-related questions, meaning BRFSS data on cannabis use was lacking for more than half of the US adult population. In the Youth Risk Behavior Surveillance System, 36 states asked standard cannabis questions; most other states had at least 1 question related to cannabis use and frequency.

**Conclusions and Relevance:**

These findings of limitations of existing surveys, and particularly the lack of national questions in the BRFSS and Youth Risk Behavior Surveillance System, suggest that available data have substantial limitations for monitoring cannabis use. As cannabis policy changes continue, there is a need to remain focused on the availability of high-quality data sources that allow for critical public health research.

## Key Points

**Question:**

Are US national data measuring aspects of cannabis use consistently available across states and over time?

**Findings:**

This case series study examining 7 state and US nationwide surveys found that limited data are available at the state or national level to monitor 8 key dimensions of cannabis use and perceptions.

**Meaning:**

These findings suggest that despite growing public support for cannabis legalization, monitoring public health effects of state and national policies may be difficult owing to a lack of available data in existing population surveys.

## Teaser

This case series study examines the types of survey data available on cannabis use among aming US adults and adolescents.

## Introduction

As of December 2019, 11 states and the District of Columbia (DC) have legalized cannabis for adult nonmedical use^1^ and 33 states and DC have legalized cannabis for medical purposes.^2^ In response to growing public support^3^ for cannabis legalization, understanding the effects of state and federal policy changes related to cannabis legalization on patterns of cannabis use is important.^4^ There is intense policy interest, including by regulators, in monitoring a number of indicators associated with cannabis use behaviors and perceptions among adolescents and adults to detect, and if necessary reduce, any negative public health effects of cannabis legalization.^5,6,7^ A challenge for monitoring and research is significant variation in data availability across and within states and over time,^4,7,8,9^ including availability of prelegalization vs postlegalization data.

Variation in available data across states highlights the need to better understand the extent of existing data in measuring effects of state and federal policy changes associated with cannabis legalization. Given ongoing policy and regulatory changes (eg, retail markets, packaging, labeling),^5,7,10^ it is critical to understand whether data are available to monitor a number of cannabis use indicators associated with specific legislative or regulatory provisions. These provisions continue to evolve as states implement legalization with varying policy and regulatory structures. Specific cannabis-related behaviors of importance include frequency, location, and methods of use; these behaviors may be risk factors for problematic cannabis use and associated outcomes.^11,12,13,14,15^ Understanding data availability to monitor cannabis use in nationally comparable surveys is critical to be able to compare findings from different states,^7,9,16,17,18^

Many states with legal cannabis have statutorily required research agendas to monitor outcomes, including public health.^9,19,20^ Negative consequences associated with cannabis use among youth are well documented and include neurocognitive defects, mental health issues, risk for dependence, lower educational attainment,^21,22,23^ and e-cigarette or vaping product use–associated lung injury.^24,25,26^ Methods of cannabis use, including among youth, are changing. A decreasing number of individuals who use cannabis report smoking, and an increasing number report use of nonsmoking modes, such as edibles, vaping, and dabbing,^27,28,29,30,31^ for which little is known about possible adverse health effects.

There is conflicting evidence about whether the prevalence of recent (ie, within the past 30 days) cannabis use increases after legalization,^16,29,32,33^ but it is clear that prevalence and frequency of use have increased significantly over the past 2 decades.^34,35^ In 2018, 45% of individuals older than 12 years had ever used marijuana and 16% used marijuana in the past year.^36^ Perceptions of risk associated with cannabis use have decreased, particularly among youth, which has been associated with a higher likelihood of future use.^3,33,37,38^

To better understand available data to monitor cannabis use among adolescents and adults, we conducted a scoping review and evaluation of survey data over time to monitor 8 indicators related to cannabis use and perceptions in Massachusetts.^39^ We also sought to inform future research and monitoring efforts by evaluating the utility of national data sources for generating state-level estimates of cannabis use and perceptions that can be compared across US states. We hypothesized that state-level data would better capture the 8 indicators over time than national data.

## Methods

We used a case series approach to identify and examine data sources available to measure cannabis use. For Massachusetts, our in-depth case study site, we analyzed data availability over time and assessed specific cannabis use questions for the general adult population as well as specific at-risk populations, including adolescents. We focused on Massachusetts because the legislation authorizing the legal retail cannabis market outlined annual monitoring requirements, but the extent of data availability to meet those requirements was unknown.^39^ We then complemented this in-depth assessment of Massachusetts data with a review of cannabis use questions in major national data sources as they apply to all 50 states and Washington, DC. The analysis was conducted between February 1, 2019, and March 18, 2020. This study was determined to be not human participants research by the University of Massachusetts Human Research Protection Office. We followed the reporting guideline for case series.^40^

### Data

We used publicly available documents describing national and state-specific data sources to determine cannabis use data availability. We first conducted a scoping review in Massachusetts to determine available data sources to measure cannabis use at the state level for the general population and specifically for adolescents. We identified data sources using published literature, national and state government surveys, and key informant interviews with state government agencies’ staff. We used website and document review to determine specific survey questions and inclusion of specific questions over time. We used the most recent questionnaire available online (as of April 15, 2019), unless the most recent version available was administered before 2015, in which case we contacted the data provider for more information. We limited this analysis to survey data that could identify prevalence of cannabis use and/or related behaviors of interest at the state level. We excluded national surveys that could not produce state-level estimates. We also excluded surveys that captured only local (ie, within-state) estimates.

We analyzed data sets available at the national level in more detail. The Behavioral Risk Factor Surveillance System (BRFSS) is jointly conducted by the Centers for Disease Control and Prevention and state agencies annually and is designed to be representative of the noninstitutionalized adult population in a state. We first determined which states used the national marijuana module containing questions related to cannabis use, frequency of use, method of use (eg, smoking, edibles), and medical vs nonmedical use. For states that did not use the national marijuana module in the most recent year for which this information is systematically available from the Centers for Disease Control and Prevention (ie, 2017), we examined state BRFSS websites to review the most recent questionnaire (as of September 3, 2019). If the questionnaire was older than 2015, we contacted the state agency responsible for the most recent version. For the Youth Risk Behavior Surveillance Survey (YRBSS), we examined state YRBSS websites to review the most recent questionnaire (as of March 15, 2020); if the questionnaire was not available, we used the most recent results available from the state YRBSS website or the Centers for Disease Control and Prevention website.

### Statistical Analysis

We assessed which data were available in Massachusetts and recorded years available, frequency of collection, population representation, age range, number of individuals surveyed, and access information. For each data source that met the inclusion criteria, we used survey questionnaires from 2011 to the most recent year available to determine cannabis-related questions over time. We analyze 8 key indicators of cannabis use patterns and perception of interest to the Massachusetts legislature, as codified in the statute that legalized cannabis for adult use in 2017^19^; this is a robust set of indicators of interest to public health researchers and policy makers. These 8 indicators include lifetime cannabis use, age of initiation, frequency of use, location of use, method of use, source of cannabis, perceptions of cannabis, and reason for use (ie, medical vs nonmedical).

For the BRFSS and YRBSS (including high school students only), after the collection of the questionnaires, we analyzed inclusion of cannabis-related questions across states. For BRFSS, we categorized states into 4 categories reflecting whether the state (1) used the national marijuana module, (2) included state-specific questions that were at least equivalent to the national marijuana module, (3) included state-specific questions related to cannabis but did not include all questions from the national module, or (4) did not include any cannabis-related questions. We used state population estimates from the 2018 bridged-race population estimates^41^ of adults aged 20 years and older to determine national BRFSS coverage with questions related to cannabis use. For the YRBSS, we categorized states into 4 categories reflecting whether the state (1) used the marijuana questions included in the national or standard questionnaire, (2) asked at a minimum about marijuana use and frequency of use in the past 30 days, (3) other, or (4) did not conduct the YRBSS. Questions from the BRFSS marijuana module and national or standard questions from the YRBSS questionnaire are presented in eTable 1 in the Supplement. To provide further context, we examined state policies and categorized them on the basis of the legal status of cannabis (ie, legalized general adult use, legalized medical use, decriminalized, or fully illegal) as of December 2019. Maps were created using ArcMap software version 10.7.1 (Esri).

## Results

We found 7 surveys containing cannabis use information for Massachusetts residents, including national and state surveys (Table 1). We included the national BRFSS for comparison purposes, although it cannot produce estimates for cannabis use in Massachusetts. Of these 7 surveys, 3 are national surveys that can produce state-level estimates and 4 are Massachusetts-specific surveys. There is some overlap in these surveys, as the National YRBSS and Massachusetts Youth Risk Behavior Survey (MYRBS) are conducted simultaneously. Overall, these surveys represent a comprehensive age range, with several adult-specific surveys, several adolescent-specific surveys, plus the National Survey on Drug Use and Health (NSDUH), which includes information on individuals 12 years and older, and the National Health and Nutrition Examination Survey (NHANES), which includes information on all ages.

**Table 1.** Summary of Survey Data Available to Monitor Cannabis Use Patterns, Perceptions, and Methods of Consumption

Data name	Source	Population included	Age range, y	Individuals, No.	Years (frequency)	Access (cost)
**National data with MA indicator**						
BRFSS	CDC	Civilian, noninstitutionalized US residents	≥18	400 000/y	1984-2017 (annually)	Public use (free)
NHANES	CDC	Civilian, noninstitutionalized US residents	All	5000/y	1999-2016 (annually, with 2 y panels)	Public use (free)^a^
NSDUH	Substance Abuse and Mental Health Services Administration	Civilian, noninstitutionalized US residents	≥12	65 000/y	1971-2017 (annually)	Public use (free)^a^
YRBSS	CDC	Middle and high school students^d^	11-18	15 000/y	1990-2017 (odd-numbered years)	Public use (free)^b^
**MA-only data**						
MA BRFSS	CDC and MA DPH	Civilian, noninstitutionalized MA residents	≥18	7000/y	1984-2017 (annually)	Limited use (free)^c^
MA Marijuana Baseline Health Study	MA DPH	Noninstitutionalized MA residents	≥18	3000	2017 (one time)	No information available
MYHS	MA DPH and DESE	MA middle and high school students	11-18	5500/y	2007-2017 (odd numbered years)	Limited use (free)^c^
MYRBS	MA DESE and CDC	MA high school students	13-18	3300/y	2007-2017 (odd numbered years)	Limited use (free)^c^

Abbreviations: BRFSS, Behavioral Risk Factor Surveillance System; CDC, Centers for Disease Control and Prevention; DESE, Department of Elementary and Secondary Education; DPH, Department of Public Health; MA, Massachusetts; MYHS, Massachusetts Youth Health Survey; MYRBS, Massachusetts Youth Risk Behavior Survey; NHANES, National Health and Nutrition Examination Survey; NSDUH, National Survey on Drug Use and Health; YRBSS, National Youth Risk Behavior Surveillance Survey.

^a^ Limited use at Research Data Center required for state indicators and drug use questions for children.

^b^ State level data are available directly from the CDC in 37 states; 10 states require obtaining data directly from state agencies conducting the survey through a variety of application processes.

^c^ Requires application process.

^d^ Only includes students in MA high schools.

Our review of detailed survey information to monitor cannabis use over time found that these surveys capture a wide variety of items and time periods, but no survey captures all 8 indicators of interest simultaneously (Table 2). The NHANES and the NSDUH include the most information for adolescents and adults, as they capture time series information about lifetime use, age of initiation, and frequency of use using standard questionnaires administered nationally. The NSDUH also captures information over time related to the source of marijuana, perceptions of marijuana, and reason for use. Although the national BRFSS marijuana module captures some of this information systematically, Massachusetts and 38 other states do not use this module.

**Table 2.** Availability of Survey Data to Monitor Cannabis Use by Data Source

Data name	Lifetime use	Age of initiation	Marijuana use			Source of marijuana	Perceptions of marijuana	Medical vs nonmedical use
			Frequency	Location	Method			
**National data with MA indicator**								
BRFSS	NA	NA	2016-2018^a^^,^^b^	NA	2016-2018^a^	NA	NA	2016-2018^a^
NHANES	2011-2016	2011-2016	2011-2016	NA	NA	NA	NA	NA
NSDUH	2011-2017	2011-2017	2011-2017	NA	NA	2011-2017	2011-2017	2013-2017
YRBSS^c^	2011-2017	2011-2017	2011-2017	2011	2015	NA	NA	NA
**MA-only data**								
Massachusetts BRFSS^d^	2015-2017^e^	NA	NA	NA	NA	NA	NA	2015-2017
MA Marijuana Baseline Health Study	NA	NA	2017	NA	2017	NA	2017	2017
MYHS	2011-2017	2011-2017	NA	NA	NA	NA	2011-2017	NA
MYRBS^f^	2013-2017	2013-2017	2013-2017	2013-2017	NA	NA	2017	NA

Abbreviations: BRFSS, Behavioral Risk Factor Surveillance System; MA, Massachusetts; MYHS, Massachusetts Youth Health Survey; MYRBS, Massachusetts Youth Risk Behavior Survey; NA, not available; NHANES, National Health and Nutrition Examination Survey; NSDUH, National Survey on Drug Use and Health; YRBSS, National Youth Risk Behavior Surveillance Survey.

^a^ Included in an optional module.

^b^ MA does not use this module.

^c^ Availability over time is based on questions available in the national and standard questionnaires.

^d^ Includes questions about marijuana use over time but does not ask questions consistently outside of the noted periods.

^e^ Does not includes questions explicitly about lifetime marijuana use but asks about use in past year.

^f^ Includes questions about problematic use but not about frequency of use.

State-specific survey data are available to fill some information gaps, although these surveys are not available over a long period, particularly in the prelegalization or premedical cannabis periods. The Massachusetts BRFSS includes questions related to problematic cannabis use; however, there were a number of changes in the questions related to cannabis over time, which makes it difficult to examine changes in behavior over time (eTable 2 in the Supplement). The Massachusetts Marijuana Baseline Health Study, conducted in 2017, captured baseline frequency of use, method of use, perceptions of marijuana, and reason for use,^42^ similar to the information captured by the national BRFSS module; however, it is unknown to what extent these measures will be assessed over time, as Massachusetts state agencies have not publicly disclosed plans to repeat this survey.

The BRFSS is a powerful national survey owing to its size, time scale, and the frequency of administration, which may allow for within- and across-state estimates; we analyzed this survey to determine which states consistently capture cannabis use data (Figure; eFigure in the Supplement). Of 50 states and DC, we found that 11 states used the national marijuana module as of 2017 (plus 1 additional state in 2018), 4 states did not use the national marijuana module but asked questions that were at least equivalent to the national module, 9 states asked any state-specific marijuana questions, and 27 states did not ask any marijuana questions (Table 3). Using state population estimates, we found that 32.2% of the US population is covered by the national marijuana module or equivalent state-specific questions.

**Figure.**
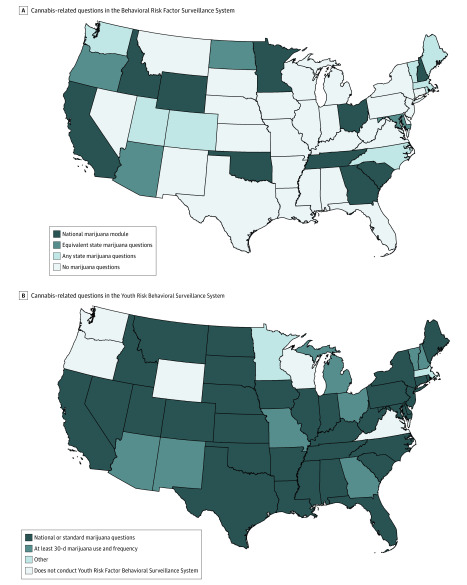
Cannabis Questions Available in National Surveys A, The most recent questionnaire available used to determine questions. Alaska and Hawaii not shown; Alaska used the national marijuana module, and Hawaii did not have cannabis-related questions. Ohio added the national marijuana module in 2018. B, Other includes the Massachusetts questionnaire, which includes a question related to 30-day use of marijuana, but does not include 30-day frequency of use; 7 states (ie, Alabama, Georgia, Indiana, Mississippi, New Jersey, Ohio, South Dakota) did not achieve adequate response rates to report population weighted estimates in 2017.

**Table 3.** Summary of Behavioral Risk Factor Surveillance System State-Level Use of Cannabis Related Questions

Category of questions	National population covered, %	States, No.^a^				
		Overall	Legalized		Decriminalized	Fully illegal
			General adult use	Medical use^b^		
National Marijuana Module	26.6	11	2	3	1	5
Equivalent state marijuana questions	5.6	4	1	3	0	0
Any state marijuana questions	11.5	9	6	2	0	1
No marijuana questions	56.3	27	3	13	2	9

^a^ States were classified by the most permissive aspect of their cannabis policy.

^b^ Legal medical use includes the use and/or sales of at least some product or plant matter containing delta-9-tetrahydrocannabinol. Seven states with legal medical cannabis have not decriminalized possession of small amounts (ie, Arkansas, Louisiana, Montana, New Jersey, Oklahoma, Pennsylvania, Utah).

For adolescents, the YRBSS, MYRBS, and Massachusetts Youth Health Survey are conducted among Massachusetts high school students (and middle school students for the Massachusetts Youth Health Survey). These are conducted in public schools every 2 years and representative of a large but select group of adolescents. These surveys capture information about lifetime use, age of initiation, frequency of use (not among middle school students), perceptions of marijuana, and location of use specific to school property. Intermittent information is available on method of use, but this has not been captured systematically over time. We analyzed questions in YRBSS state questionnaires and found that 36 states ask questions consistent with the standard or national questions in the most recent year (Figure, B; eFigure in the Supplement). Substantial variation exists in state level questionnaires, with 6 states asking questions related to mode of use, 6 states asking questions related to perceptions of use, and 3 states asking about use on school property, among others. In 2017, 7 states did not have adequate response rates to report population-weighted estimates.

## Discussion

This case series study found a number of sources that could be used to monitor cannabis use over time, but many of these data sources have substantial limitations that restrict their usefulness in understanding effects of state and federal policy changes. Our results highlight gaps in state data collection and how these gaps affect knowledge surrounding the public health effects of cannabis legalization.

Contrary to our hypothesis, we found that Massachusetts—which legalized medical cannabis in 2012 and general adult use of cannabis in 2016—does not have consistently available state data sources to monitor all 8 key cannabis use indicators of interest and that state-level data were not as robust as national data sources. Despite the public health importance of maintaining nationally comparable survey data over time to allow for outcomes analyses of anticipated and unanticipated policy changes,^9^ our results show this is not happening consistently within Massachusetts or using the BRFSS and YRBSS nationally. We found that although 2 major national surveys, the NSDUH and NHANES, can provide substantial information about multiple dimensions of cannabis use over time, these surveys are limited by sample size in their ability to make across- and within-state comparisons, particularly at the annual level. This is an important limitation when one considers the rapid pace at which legalization and related policies are changing and the need to detect potential changes in key indicators in as timely a manner as possible.

Monitoring cannabis use among adolescents is of particular public health importance owing to adverse effects associated with cannabis use on brain development, potential substance use dependence later in life, and changes in perceptions around cannabis use and changing methods of use.^3,27,28,29,30,31,33,37,38^ In Massachusetts, we found that there are national and state-specific surveys available (under restricted access policies from data providers) that monitor cannabis use and frequency; however, these surveys have not consistently collected information over time about method of use, location of use, and/or perceptions of cannabis, all of which are important to fully quantify changing patterns of use over time. As cannabis is legalized for adult use, monitoring diversion of legal cannabis into the adolescent population is particularly important. These youth surveys, available in Massachusetts, are not available in all states with medical or nonmedical cannabis.^9^ Colorado is a leader in carefully tracking these measures. Colorado was one of the first states to legalize adult use of cannabis and has carefully tracked the public health implications of legalization, including through the addition of questions over time to the Healthy Kids Colorado Survey, such that the most recent version included questions on 6 of the 8 dimensions we examine (ie, lifetime use, age of initiation, location of use, method of use, source of marijuana, and perceptions of marijuana).^43^

Understanding the availability and comparability of BRFSS data across states with respect to cannabis-related questions is a novel contribution of our analysis. The NSDUH is able to make state-specific estimates at the annual level using restricted data or using 2-year periods using public use data; additional national data sources are necessary to research the associations of time-sensitive state policy changes and make within-state comparisons. The BRFSS has a much larger sample size at the state level, but we found that a large number of states, with more than 56.3% of the national population, do not include any cannabis use questions. The national marijuana module became available for use in 2016; in early legalization states (ie, states that legalized cannabis before 2016), the national module does not contribute substantial information for understanding changes in cannabis use during longer periods or across the prelegalization to postlegalization transition. However, if implemented in states prior to legalization for adult use, the national BRFSS marijuana module would likely provide useful data.^3,9^ This shows the importance of changes to BRFSS core questions, including adding questions on cannabis use, in a rapidly changing policy environment.^3,4^ This is particularly important to monitor outcomes among at-risk population groups, such as adolescents, persons with substance use or mental health disorders, and pregnant women, who may be disproportionately affected by cannabis legalization and for whom there is likely not adequate sample size to examine carefully at the state level in national surveys. Additionally, to our knowledge, no data are available to measure cannabis use among incarcerated populations, who have high rates of substance use disorders.^44^

### Limitations

Our study has several limitations. The first limitation is that our primary analysis of available surveys was limited to Massachusetts; as we show with variation in availability in the BRFSS and YRBSS, substantial differences in data availability exist across states, so data available in Massachusetts may not be generalizable to the rest of the country. However, this in-depth analysis of data available for Massachusetts is an important case study to understand what may be available in other states and reveals issues likely to be common across states. The second limitation is that we limited our analysis to survey data, as this is the most commonly used way of obtaining prevalence estimates for cannabis use.^9^ Administrative or other data sources, such as health insurance claims data or substance use treatment facility discharge data,^45^ may also capture changes in cannabis use associated with cannabis use disorder. Owing to the very small number of surveys measuring polysubstance use, we were not able to consider concurrent use of cannabis and other substances, such as alcohol. This is an important extension in considering future data collection, particularly since little is known about polysubstance use for adults and adolescents.^46,47^ Third, because we included only data sources that support estimation at the state level, we excluded a number of data sources with potential relevance to research on cannabis policy (eg, the American College Health Survey and Monitoring the Future). Fourth, our focus on the general population omitted some important subgroups, such as pregnant women, for whom there is growing interest and concern regarding cannabis use.

## Conclusions

In this case series study, we report 2 large, high quality, nationally representative surveys—NHANES and NSDUH—that can be used to monitor cannabis use and the outcomes associated with state and national policy changes; however, substantial challenges remain in the availability of data needed to fully monitor the public health effects associated with cannabis legalization. Existing surveys, and particularly the lack of national core questions in the BRFSS or standardized required questions in the YRBSS, suggest that available data have substantial limitations for monitoring cannabis use along the 8 dimensions we examined. As research into the effects of cannabis legalization and other changes in cannabis policy continue, remaining focused on the availability of high-quality data sources will allow critical public health research.

## Notes

Supplement. **eFigure.** Cannabis Questions Available in the Behavioral Risk Factor Surveillance System and Youth Risk Behavior Surveillance Survey and State Cannabis Legalization Status **eTable 1.** Cannabis Use Questions in Behavioral Risk Factor Surveillance System and Youth Risk Behavior Surveillance Survey **eTable 2.** Cannabis Use Questions in Massachusetts Behavioral Risk Factor Surveillance System from 2011-2017

## References

[1] National Conference of State Legislatures Marijuana overview. Accessed August 7, 2019. https://www.ncsl.org/research/civil-and-criminal-justice/marijuana-overview.aspx

[2] National Conference of State Legislatures State medical marijuana laws. Accessed August 7, 2019. https://www.ncsl.org/research/health/state-medical-marijuana-laws.aspx

[3] Cohn AM, Johnson AL, Rose SW, Rath JM, Villanti AC Support for marijuana legalization and predictors of intentions to use marijuana more often in response to legalization among U.S. young adults. Subst Use Misuse. 2017;52(2):203-213. doi:10.1080/10826084.2016.1223688 27976988

[4] Spetz J, Chapman SA, Bates T, Jura M, Schmidt LA Social and political factors associated with state-level legalization of cannabis in the United States. Contemp Drug Probl. 2019;46(2):165-179. doi:10.1177/0091450919827605 PMC8022331 33828345

[5] Pacula RL, Kilmer B, Wagenaar AC, Chaloupka FJ, Caulkins JP Developing public health regulations for marijuana: lessons from alcohol and tobacco. Am J Public Health. 2014;104(6):1021-1028. doi:10.2105/AJPH.2013.301766 24825201 PMC4062005

[6] Ghosh T, Van Dyke M, Maffey A, Whitley E, Gillim-Ross L, Wolk L The public health framework of legalized marijuana in Colorado. Am J Public Health. 2016;106(1):21-27. doi:10.2105/AJPH.2015.302875 26562117 PMC4695936

[7] Pacula RL, Smart R Medical marijuana and marijuana legalization. Annu Rev Clin Psychol. 2017;13:397-419. doi:10.1146/annurev-clinpsy-032816-045128 28482686 PMC6358421

[8] Dilley JA, Hitchcock L, McGroder N, Greto LA, Richardson SM Community-level policy responses to state marijuana legalization in Washington state. Int J Drug Policy. 2017;42:102-108. doi:10.1016/j.drugpo.2017.02.010 28365192 PMC5473373

[9] Binkin N CSTE Marijuana Surveillance: Environmental Scan Report 2018. Council of State and Territorial Epidemiologists; 2018.

[10] MacCoun RJ, Mello MM Half-baked—the retail promotion of marijuana edibles. N Engl J Med. 2015;372(11):989-991. doi:10.1056/NEJMp1416014 25760351

[11] Baggio S, Deline S, Studer J, Mohler-Kuo M, Daeppen JB, Gmel G Routes of administration of cannabis used for nonmedical purposes and associations with patterns of drug use. J Adolesc Health. 2014;54(2):235-240. doi:10.1016/j.jadohealth.2013.08.013 24119417

[12] Adamson SJ, Kay-Lambkin FJ, Baker AL, An improved brief measure of cannabis misuse: the Cannabis Use Disorders Identification Test-Revised (CUDIT-R). Drug Alcohol Depend. 2010;110(1-2):137-143. doi:10.1016/j.drugalcdep.2010.02.017 20347232

[13] Cuttler C, Spradlin A Measuring cannabis consumption: psychometric properties of the Daily Sessions, Frequency, Age of Onset, and Quantity of Cannabis Use Inventory (DFAQ-CU). PLoS One. 2017;12(5):e0178194. doi:10.1371/journal.pone.0178194 28552942 PMC5446174

[14] Beck KH, Caldeira KM, Vincent KB, O’Grady KE, Wish ED, Arria AM The social context of cannabis use: relationship to cannabis use disorders and depressive symptoms among college students. Addict Behav. 2009;34(9):764-768. doi:10.1016/j.addbeh.2009.05.001 19497678 PMC2709927

[15] Swift W, Coffey C, Carlin JB, Degenhardt L, Patton GC Adolescent cannabis users at 24 years: trajectories to regular weekly use and dependence in young adulthood. Addiction. 2008;103(8):1361-1370. doi:10.1111/j.1360-0443.2008.02246.x 18855826

[16] Cerdá M, Wall M, Feng T, Association of state recreational marijuana laws with adolescent marijuana use. JAMA Pediatr. 2017;171(2):142-149. doi:10.1001/jamapediatrics.2016.3624 28027345 PMC5365078

[17] Cerdá M, Wall M, Keyes KM, Galea S, Hasin D Medical marijuana laws in 50 states: investigating the relationship between state legalization of medical marijuana and marijuana use, abuse and dependence. Drug Alcohol Depend. 2012;120(1-3):22-27. doi:10.1016/j.drugalcdep.2011.06.011 22099393 PMC3251168

[18] Dai H, Richter KP A national survey of marijuana use among US adults with medical conditions, 2016-2017. JAMA Netw Open. 2019;2(9):e1911936. doi:10.1001/jamanetworkopen.2019.11936 31539078 PMC6755533

[19] Mass Sess Law. An Act To Ensure Safe Access To Marijuana. ch 55 (2017).

[20] Reed J; Colorado Division of Criminal Justice Impacts of marijuana legalization in Colorado: a report pursuant to Senate Bill 13-283. Updated 2018 Accessed August 7, 2019. https://cdpsdocs.state.co.us/ors/docs/reports/2018-SB13-283_Rpt.pdf

[21] D’Amico EJ, Rodriguez A, Tucker JS, Pedersen ER, Shih RA Planting the seed for marijuana use: changes in exposure to medical marijuana advertising and subsequent adolescent marijuana use, cognitions, and consequences over seven years. Drug Alcohol Depend. 2018;188:385-391. doi:10.1016/j.drugalcdep.2018.03.031 29779761 PMC6744951

[22] Graves JM, Whitehill JM, Miller ME, Brooks-Russell A, Richardson SM, Dilley JA Employment and marijuana use among Washington State adolescents before and after legalization of retail marijuana. J Adolesc Health. 2019;65(1):39-45. doi:10.1016/j.jadohealth.2018.12.027 30879883 PMC6589368

[23] Weiss SRB, Howlett KD, Baler RD Building smart cannabis policy from the science up. Int J Drug Policy. 2017;42:39-49. doi:10.1016/j.drugpo.2017.01.007 28189459 PMC5404989

[24] Centers for Disease Control and Prevention Outbreak of lung injury associated with the use of e-cigarette, or vaping, products. Updated February 2020 Accessed March 17, 2020. https://www.cdc.gov/tobacco/basic_information/e-cigarettes/severe-lung-disease.html

[25] Smith DM, Goniewicz ML The role of policy in the EVALI outbreak: solution or contributor? Lancet Respir Med. 2020;8(4):343-344. doi:10.1016/S2213-2600(20)30065-5 32043987

[26] Navon L, Jones CM, Ghinai I, Risk factors for e-cigarette, or vaping, product use-associated lung injury (EVALI) among adults who use e-cigarette, or vaping, products—Illinois, July-October 2019. MMWR Morb Mortal Wkly Rep. 2019;68(45):1034-1039. doi:10.15585/mmwr.mm6845e1 31725708 PMC6855514

[27] Borodovsky JT, Crosier BS, Lee DC, Sargent JD, Budney AJ Smoking, vaping, eating: is legalization impacting the way people use cannabis? Int J Drug Policy. 2016;36:141-147. doi:10.1016/j.drugpo.2016.02.022 26992484 PMC5010515

[28] Borodovsky JT, Lee DC, Crosier BS, Gabrielli JL, Sargent JD, Budney AJUS U.S. cannabis legalization and use of vaping and edible products among youth. Drug Alcohol Depend. 2017;177:299-306. doi:10.1016/j.drugalcdep.2017.02.017 28662974 PMC5534375

[29] Dilley JA, Richardson SM, Kilmer B, Pacula RL, Segawa MB, Cerdá M Prevalence of cannabis use in youths after legalization in Washington state. JAMA Pediatr. 2019;173(2):192-193. doi:10.1001/jamapediatrics.2018.4458 30566196 PMC6439594

[30] Russell C, Rueda S, Room R, Tyndall M, Fischer B Routes of administration for cannabis use—basic prevalence and related health outcomes: a scoping review and synthesis. Int J Drug Policy. 2018;52:87-96. doi:10.1016/j.drugpo.2017.11.008 29277082

[31] Knapp AA, Lee DC, Borodovsky JT, Auty SG, Gabrielli J, Budney AJ Emerging trends in cannabis administration among adolescent cannabis users. J Adolesc Health. 2019;64(4):487-493. doi:10.1016/j.jadohealth.2018.07.012 30205931 PMC6408312

[32] Estoup AC, Moise-Campbell C, Varma M, Stewart DG The impact of marijuana legalization on adolescent use, consequences, and perceived risk. Subst Use Misuse. 2016;51(14):1881-1887. doi:10.1080/10826084.2016.1200623 27612596

[33] Miech R, Johnston L, O’Malley PM Prevalence and attitudes regarding marijuana use among adolescents over the past decade. Pediatrics. 2017;140(6):e20170982. doi:10.1542/peds.2017-0982 29109106 PMC5703791

[34] Chawla D, Yang YC, Desrosiers TA, Westreich DJ, Olshan AF, Daniels JL Past-month cannabis use among U.S. individuals from 2002-2015: an age-period-cohort analysis. Drug Alcohol Depend. 2018;193:177-182. doi:10.1016/j.drugalcdep.2018.05.035 30384326 PMC6542262

[35] Volkow ND, Han B, Compton WM, McCance-Katz EF Self-reported medical and nonmedical cannabis use among pregnant women in the United States. JAMA. 2019;322(2):167-169. doi:10.1001/jama.2019.7982 31211824 PMC6582258

[36] Substance Abuse and Mental Health Services Administration National Survey on Drug Use and Health, 2018. Accessed March 17, 2020. https://pdas.samhsa.gov/#/survey/NSDUH-2018-DS0001

[37] Maxwell JC, Mendelson B What do we know now about the impact of the laws related to marijuana? J Addict Med. 2016;10(1):3-12. doi:10.1097/ADM.0000000000000188 26818826 PMC4733622

[38] Sarvet AL, Wall MM, Keyes KM, Recent rapid decrease in adolescents’ perception that marijuana is harmful, but no concurrent increase in use. Drug Alcohol Depend. 2018;186:68-74. doi:10.1016/j.drugalcdep.2017.12.041 29550624 PMC6134844

[39] Whitehill J, Geissler K, Doonan S, Johnson J Special Report: Evaluating the Impact of Cannabis Legalization in Massachusetts—State of the Data. Massachusetts Cannabis Control Commission; 2019.

[40] Kempen JH Appropriate use and reporting of uncontrolled case series in the medical literature. Am J Ophthalmol. 2011;151(1):7-10.e1. doi:10.1016/j.ajo.2010.08.047 21163373 PMC3052978

[41] United States Department of Health and Human Services, Centers for Disease Control and Prevention, National Center for Health Statistics CDC WONDER Online Database: bridged-race population estimates 1990-2018 request. Accessed September 4, 2019. https://wonder.cdc.gov/bridged-race-v2018.html

[42] Massachusetts Department of Public Health Marijuana baseline health study: final report—July 2019. Accessed September 4, 2019. https://www.mass.gov/files/documents/2019/07/09/MBHS-full-report-final.pdf

[43] Colorado Department of Public Health and Environment Healthy Kids Colorado Survey data tables and reports. Accessed September 23, 2019. https://www.colorado.gov/pacific/cdphe/healthy-kids-colorado-survey-data-tables-and-reports

[44] Fazel S, Yoon IA, Hayes AJ Substance use disorders in prisoners: an updated systematic review and meta-regression analysis in recently incarcerated men and women. Addiction. 2017;112(10):1725-1739. doi:10.1111/add.13877 28543749 PMC5589068

[45] Geissler KH. Johnson JK, Whitehill JM Utility of survey and administrative data for understanding health impacts of policy changes related to substance use. Oral presentation at: the Annual Conference of the American Society for Health Economics; June 23-26, 2019; Washington, DC.

[46] Connor JP, Gullo MJ, Chan G, Young RM, Hall WD, Feeney GF Polysubstance use in cannabis users referred for treatment: drug use profiles, psychiatric comorbidity and cannabis-related beliefs. Front Psychiatry. 2013;4:79. doi:10.3389/fpsyt.2013.00079 23966956 PMC3736050

[47] Connor JP, Gullo MJ, White A, Kelly AB Polysubstance use: diagnostic challenges, patterns of use and health. Curr Opin Psychiatry. 2014;27(4):269-275. doi:10.1097/YCO.0000000000000069 24852056

