# Supplementary material for: Evaluation of Availability of Survey Data About Cannabis Use
Source: JAMA Netw Open. 2020 Jun 10;3(6):e206039. doi: 10.1001/jamanetworkopen.2020.6039 (PMC7287570; doi:10.1001/jamanetworkopen.2020.6039)
Supplement: Supplement. — eFigure. Cannabis Questions Available in the Behavioral Risk Factor Surveillance System and Youth Risk Behavior Surveillance Survey and State Cannabis Legalization Status eTable 1. Cannabis Use Questions in Behavioral Risk Factor Surveillance System and Youth Risk Behavior Surveillance Survey eTable 2. Cannabis Use Questions in Massachusetts Behavioral Risk Factor Surveillance System from 2011-2017 [file jamanetwopen-3-e206039-s001.pdf]

Supplementary Online Content

Geissler KH, Krazier K, Johnson JK, Doonan SM, Whitehill JM. Evaluation of availability of survey data about cannabis use. *JAMA Netw Open*. 2020;3(6)e206039. doi:10.1001/jamanetworkopen.2020.6039

eFigure. Cannabis Questions Available in the Behavioral Risk Factor Surveillance System and Youth Risk Behavior Surveillance Survey and State Cannabis Legalization Status
eTable 1. Cannabis Use Questions in Behavioral Risk Factor Surveillance System and Youth Risk Behavior Surveillance Survey
eTable 2. Cannabis Use Questions in Massachusetts Behavioral Risk Factor Surveillance System from 2011-2017

This supplementary material has been provided by the authors to give readers additional information about their work.

© 2020 Geissler KR et al. *JAMA Network Open.*

eFigure. Cannabis Questions Available in the Behavioral Risk Factor Surveillance System and Youth Risk Behavior Surveillance Survey and State Cannabis Legalization Status

Panel A: Cannabis questions available in Behavioral Risk Factor Surveillance System, with state cannabis legalization status

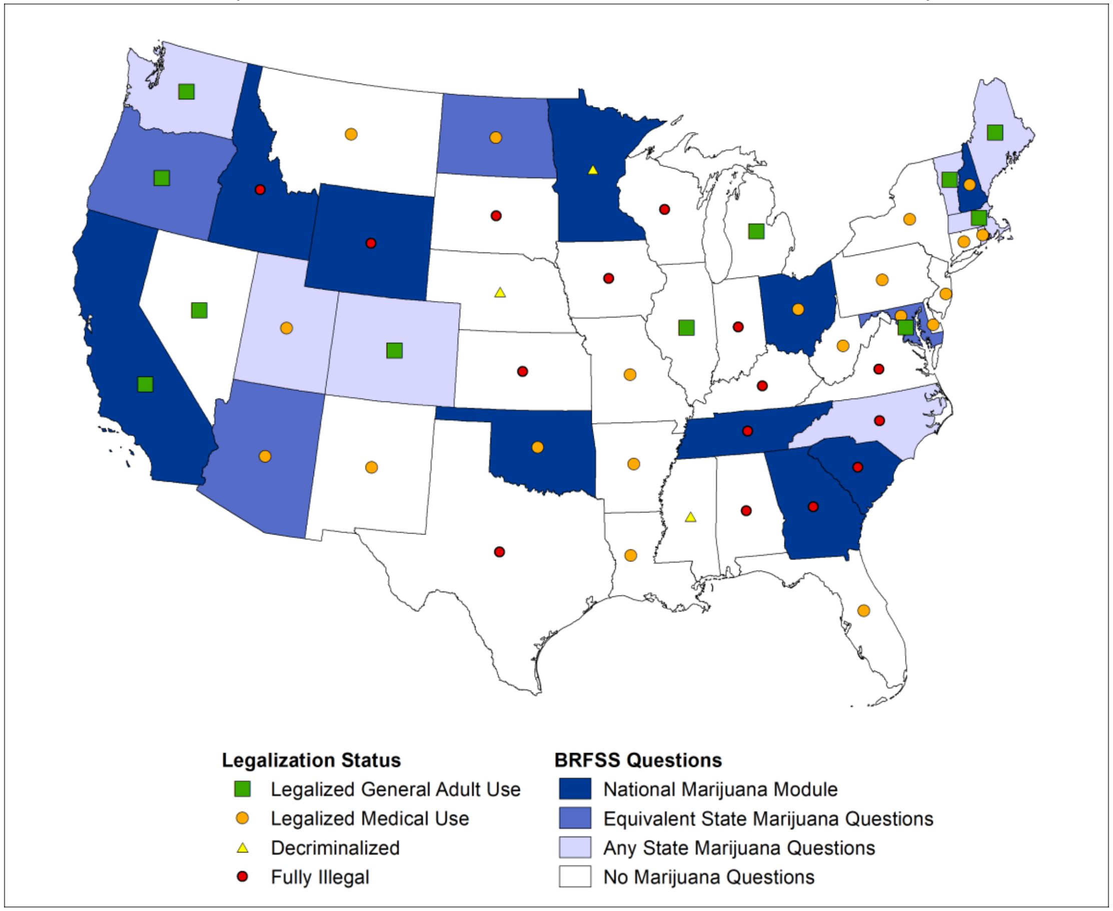

Note: Most recent questionnaire available used to determine questions. Alaska and Hawaii not shown. Alaska used the national marijuana module. Hawaii did not have cannabis-related questions. Ohio added the national marijuana module in 2018. States were classified by the most permissive aspect of their cannabis policy. Legal medical use includes the use and/or sales of at least some product or plant matter containing delta-9-tetrahydrocannabinol. Seven states with legal medical cannabis have not decriminalized possession of small amounts (ie, Arkansas, Louisiana, Montana, New Jersey, Oklahoma, Pennsylvania, Utah).

© 2020 Geissler KR et al. *JAMA Network Open.*

Panel B: Cannabis questions available in Youth Risk Behavior Surveillance Survey, with state cannabis legalization status

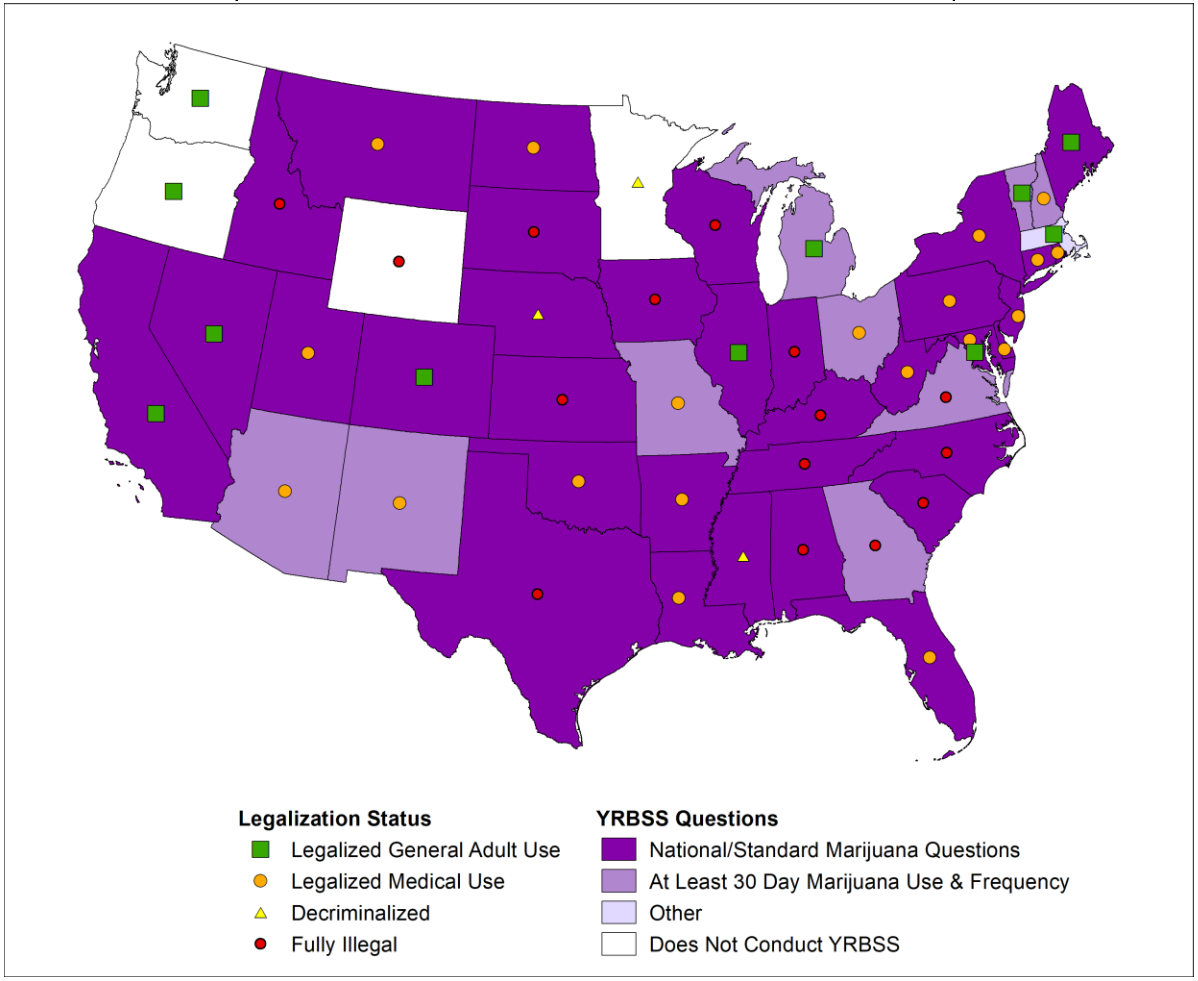

Note: The most recent questionnaire available used to determine questions. Alaska and Hawaii not shown; Alaska used the national marijuana module, and Hawaii did not have cannabis-related questions. Ohio added the national marijuana module in 2018. States were classified by the most permissive aspects of their cannabis policy. Legal medical use includes the use and/or sales of at least some product or plant matter containing delta-9-tetrahydrocannabinol. Seven states with legal medical cannabis have not decriminalized posession of small amounts (ie, Arkansas, Louisiana, Montana, New Jersey, Oklahoma, Pennsylvania, Utah).

© 2020 Geissler KR et al. *JAMA Network Open.*

eTable 1. Cannabis Use Questions in Behavioral Risk Factor Surveillance System and Youth Risk Behavior Surveillance Survey

	Question	Response Options
Panel A: Behavioral Risk Factor Surveillance System national marijuana module		
1	During the past 30 days, on how many days did you use marijuana or hashish?	• _ _ (1-30) Number of Days<br>• None (0 days) [Go to Next Module]<br>• Don't know/not sure [Go to Next Module]<br>• Refused [Go to Next Module]
2	During the past 30 days, what was the primary mode you used marijuana? Please select one. Did you... [Note: Asked Only of Current Marijuana Users]	• Smoke it, for example, in a joint, bong, pipe, or blunt.<br>• Eat it, for example, in brownies, cakes, cookies, or candy.<br>• Drink it, for example, in tea, cola, or alcohol.<br>• Vaporize it, for example, in an e-cigarette-like vaporizer or another vaporizing device.<br>• Dab it, for example, using waxes or concentrates.<br>• Use it some other way.<br>• Don't know/not sure [Go to Next Module]<br>• Refused
3	When you used marijuana or hashish during the past 30 days, was it for medical reasons to treat or decrease symptoms of a health condition, or was it for non-medical reasons to get pleasure or satisfaction (such as: excitement, to "fit in" with a group, increased awareness, to forget worries, for fun at a social gathering). [Note: Asked Only of Current Marijuana Users].	• Only for medical reasons to treat or decrease symptoms of a health condition<br>• Only for non-medical purposes to get pleasure or satisfaction<br>• Both medical and non-medical reasons<br>• Don't know/Not sure<br>• Refused
Panel B: Youth Risk Behavior Surveillance Survey standard/national marijuana questions		
1	During your life, how many times have you used marijuana?	• 0 times<br>• 1 or 2 times<br>• 3 to 9 times<br>• 10 to 19 times<br>• 20 to 39 times

© 2020 Geissler KR et al. *JAMA Network Open.*

		• 40 to 99 times<br>• 100 or more times
2	How old were you when you tried marijuana for the first time?	• I have never tried marijuana<br>• 8 years old or younger<br>• 9 or 10 years old<br>• 11 or 12 years old<br>• 13 or 14 years old<br>• 15 or 16 years old<br>• 17 years old or older
3	During the past 30 days, how many times did you use marijuana?	• 0 times<br>• 1 or 2 times<br>• 3 to 9times<br>• 10 to 19 times<br>• 20 to 39 times<br>• 40 or more times

© 2020 Geissler KR et al. *JAMA Network Open.*

eTable 2. Cannabis Use Questions in Massachusetts Behavioral Risk Factor Surveillance System from 2011-2017

Question	2011	2012[a]	2013	2014[a]	2015	2016	2017
"Non-medical" drug use means using it to get high or for pleasurable effects, see what the effects are like, or use with friends. In your lifetime, have you taken any of the following drugs six or more times for non-medical purposes?<br><br>Marijuana or hashish?<br>[If needed: Street names include: "pot," "grass," and "hash."]	X		X				
"Non-medical" drug use means using it to get high or experience pleasurable effects, see what the effects are like, or take with friends.<br><br>Have you taken the following drugs for non-medical purposes during the past year…<br><br>Marijuana or hashish?					X	X	X
Has a doctor or other health professional ever prescribed the following medicines for you to treat a medical or psychological problem…<br><br>Medical marijuana or related prescription drugs, such as Sativex, Marinol, Nabilone, or Cesamet?					X	X	X
To summarize, you said that non-medical drug use caused you _____, ______, (and) _________ (medical problems/withdrawal sickness/to reduce important activities/loss of control/to be unable to quit/to spend a lot of time on it/tolerance). The next questions are about those experiences.<br><br>I am going to read the name of (the/each) drug you used non-medically six times or more. Please tell me when it last caused you to have (three/ three or more) of these experiences within the same twelve months. The choices are: in the past year, within the past two years, more than two years ago, or never.<br><br>[Marijuana option if responded had used at least six or more times]	X						

© 2020 Geissler KR et al. *JAMA Network Open.*

Question	2011	2012[a]	2013	2014[a]	2015	2016	2017
To summarize, you said that non-medical drug use caused you to ____ and ____(be in hazardous situations, neglect responsibilities, have interpersonal problems, have legal problems). The next questions are about that experience/those experiences.<br><br>I am going to read the name of (the/each) drug you have used non-medically six or more times. Please tell me when it last caused you to have that experience/ one of those experiences. The choices are: in the past year, within the past two years, more than two years ago, or never.<br><br>[Marijuana option if responded had used at least six or more times]	X						
To summarize, you said that drug use caused you _____, ______, (and) _________ (medical problems/to reduce important activities/loss of control/to be unable to quit/to spend a lot of time on it/be in dangerous situations/neglect responsibilities/have interpersonal problems/craving for the drug/withdrawal sickness/tolerance). The next questions are about those experiences.<br><br>I am going to read the name of (the/each) drug you used non-medically six times or more. Please tell me when it last caused you to have (two/two or more) of these experiences, counting withdrawal and tolerance, within the same twelve months. Your answers can be: in the past year, more than a year ago, or never.<br><br>[Marijuana option if responded had used at least six or more times]			X				
[If yes to medical or non-medical marijuana use] Has your use of Marijuana caused problems with your physical or mental health, work or school, or family or friends in the past year?					X	X	X
In the past year, have you felt dependent on Marijuana or experienced trouble getting off of the drug when you no longer needed it medically or wanted to use it non-medically?					X	X	X
Have you gone to an emergency room, obtained medical treatment, or received professional counseling for adverse effects of your use of Marijuana in the past year?					X	X	X

Note: In many years, marijuana questions are asked to only a portion of the sample, limiting the sample size of respondents.

[a] No cannabis related questions were asked in the 2012 or 2014 surveys.

© 2020 Geissler KR et al. *JAMA Network Open.*
